# Zr_3_NiSb_7_: a new anti­mony-enriched ZrSb_2_ derivative

**DOI:** 10.1107/S1600536808020278

**Published:** 2008-07-05

**Authors:** V. Romaka, A. Tkachuk, L. Romaka

**Affiliations:** a Department of Inorganic Chemistry, Ivan Franko National University of Lviv, Kyryla i Mefodiya Street 6, 79005 Lviv, Ukraine; b Department of Chemistry, University of Alberta, Edmonton, Alberta, Canada T6G 2G2

## Abstract

Single crystals of trizirconium nickel hepta­anti­monide were synthesized from the constituent elements by arc-melting. The compound crystallizes in a unique structure type and belongs to the family of two-layer structures. All crystallographically unique atoms (3 × Zr, 1 × Ni and 7 × Sb) are located at sites with *m* symmetry. The structure contains ‘Zr_2_Ni_2_Sb_5_’ and ‘Zr_4_Sb_9_’ fragments and might be described as a new ZrSb_2_ derivative with a high Sb content.

## Related literature

The structure of ZrSb_2_ was described by Garcia & Corbett (1988 ▶). For related anti­monides, see: Romaka *et al.* (2007 ▶); Tkachuk *et al.* (2007 ▶). For related literature, see: Emsley (1991 ▶).

## Experimental

### 

#### Crystal data

Zr_3_NiSb_7_
*M* *_r_* = 1184.62
Orthorhombic, 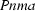

*a* = 17.5165 (19) Å
*b* = 3.9266 (4) Å
*c* = 14.3968 (15) Å
*V* = 990.22 (18) Å^3^
*Z* = 4
Mo *K*α radiation
μ = 23.56 mm^−1^
*T* = 295 (2) K
0.37 × 0.06 × 0.04 mm

#### Data collection

Bruker SMART 1000 diffractometer
Absorption correction: numerical (*SHELXTL*; Sheldrick, 2008 ▶) *T*_min_ = 0.057, *T*_max_ = 0.426
11118 measured reflections
1722 independent reflections
1500 reflections with *I* > 2σ(*I*)
*R*_int_ = 0.043

#### Refinement

*R*[*F* ^2^ > 2σ(*F* ^2^)] = 0.024
*wR*(*F* ^2^) = 0.053
*S* = 1.18
1722 reflections
68 parameters
Δρ_max_ = 2.12 e Å^−3^
Δρ_min_ = −2.89 e Å^−3^

### 

Data collection: *SMART* (Bruker, 2000 ▶); cell refinement: *SAINT-Plus* (Bruker, 2000 ▶); data reduction: *SAINT-Plus*; program(s) used to solve structure: *SHELXS97* (Sheldrick, 2008 ▶); program(s) used to refine structure: *SHELXL97* (Sheldrick, 2008 ▶); molecular graphics: *DIAMOND* (Brandenburg, 1999 ▶); software used to prepare material for publication: *SHELXL97*.

## Supplementary Material

Crystal structure: contains datablocks I, global. DOI: 10.1107/S1600536808020278/wm2182sup1.cif

Structure factors: contains datablocks I. DOI: 10.1107/S1600536808020278/wm2182Isup2.hkl

Additional supplementary materials:  crystallographic information; 3D view; checkCIF report

## Figures and Tables

**Table 1:** Selected bond lengths (Å)

Zr1—Sb4^i^	2.9620 (6)
Zr1—Sb6	2.9876 (8)
Zr1—Sb1^ii^	3.0669 (8)
Zr1—Sb3^iii^	3.0720 (7)
Zr1—Sb7^iii^	3.0960 (6)
Zr1—Sb5	3.1324 (8)
Zr2—Sb6^iv^	2.9478 (6)
Zr2—Sb4^iii^	2.9499 (6)
Zr2—Sb2^iv^	2.9604 (6)
Zr2—Sb2^ii^	3.0029 (8)
Zr2—Sb5	3.0044 (8)
Zr3—Sb1^iv^	2.9569 (6)
Zr3—Sb3^iv^	2.9944 (7)
Zr3—Sb5^iv^	2.9958 (6)
Zr3—Sb6^v^	2.9975 (8)
Zr3—Sb3^vi^	3.1616 (9)
Ni1—Sb7	2.5728 (10)
Ni1—Sb2^iv^	2.5875 (7)
Ni1—Sb1^iv^	2.6140 (7)
Ni1—Sb4^vi^	2.7141 (10)
Sb1—Sb2	3.1998 (8)
Sb1—Sb3^vii^	3.2645 (6)
Sb1—Sb4^viii^	3.2981 (6)
Sb2—Sb2^ix^	3.2250 (7)
Sb2—Sb4^viii^	3.3111 (6)
Sb5—Sb7^iii^	3.1380 (6)
Sb5—Sb6^iv^	3.1387 (6)
Sb6—Sb7^i^	3.1393 (6)

Symmetry codes: (i) 
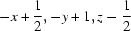
; (ii) 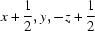; (iii) 
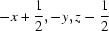
; (iv) 
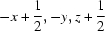
; (v) 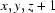; (vi) 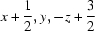; (vii) 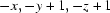; (viii) 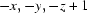; (ix) 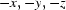.

**Fig. 1.**
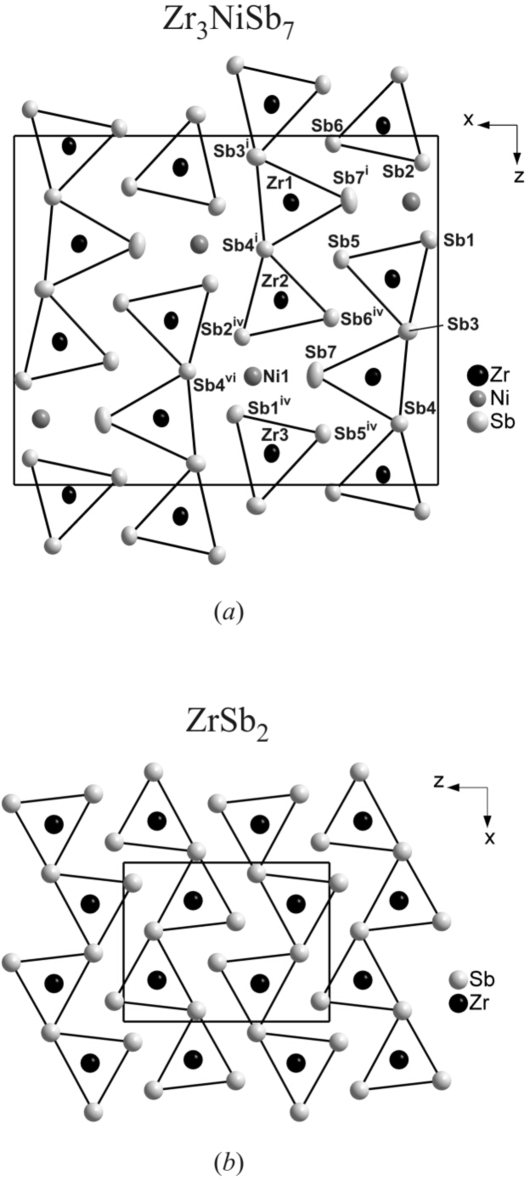
(a). Projection of the Zr3NiSb7 structure onto the (010) plane with displacement ellipsoids drawn at the 95% probability level. [Symmetry codes: (i) 0.5 - x, 1 - y, -1/2 + z; (iv) 0.5 - x, -y, 0.5 - z; (vi) 1/2 + x, y, 1.5 - z]; (b) Projection of the ZrSb2 structure onto the (010) plane.

**Fig. 2.**
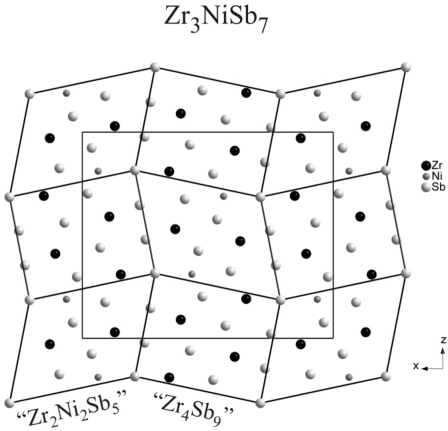
The stacked "Zr2Ni2Sb5" and "Zr4Sb9" fragments in the Zr3NiSb7 structure.

## supplementary crystallographic information

### Comment

Antimony based intermetallics attract interest due to interesting thermoelectric properties of some phases, *e.g.* antimonides with MgAgAs and Y_3_Au_3_Sb_4_ type structures. The investigation of new intermetallic phases is useful for the development of new materials, and the accurate determination of their crystal structures is a basic requirement for a better understanding of the corresponding physical properties.

Investigation of the Zr–Ni–Sb ternary system revealed the presence of several compounds in the Sb-enriched area (Romaka *et al.*, 2007), including the new title antimonide with composition Zr_27_Ni_9_Sb_64_ (in %_at_). Interatomic distances (Table 1) between Sb atoms are in good agreement with the sum of the atomic radius (Emsley, 1991), whereas the majority of Zr—Sb and all Ni—Sb distances are somewhat shortened. Such shortening may be explained by partial covalent bonding which appears to be significant between Ni—Sb atoms because their contact distances are rather close to the sum of their covalent radii (2.56 Å). As the majority of ternary intermetallics are constructed from the fragments of their most stable binary compounds, the structure analysis of the antimonides Zr_3_NiSb_7_ and the already known Zr_2_NiSb_3_ (Tkachuk *et al.*, 2007) in the Sb-enriched area shows that both can be derived from the binary compound ZrSb_2_ (Garcia & Corbett, 1988), which crystallizes in the PbCl_2_ structure type.

Zr_3_NiSb_7_ belongs to the family of two-layer structures. It may be represented as a net of trigonal prisms formed by Sb atoms that are bridged by nickel atoms (Fig. 1a). Such an arrangement is very similar to that in the binary ZrSb_2_ structure (Fig. 1b). The coordination polyhedra are distorted tri-capped trigonal prisms for the Zr atoms, and distorted octahedra for Ni atoms. In an alternative description, the Zr_3_NiSb_7_ structure contains fragments of the hypothetical "Zr_2_Ni_2_Sb_5_" and "Zr_4_Sb_9_" structures (Fig. 2) which are so far unknown for the ternary Zr–Ni–Sb or binary Zr–Sb systems. The main feature of the Zr_3_NiSb_7_ structure is the absence of covalent bonding between antimony atoms in contrast to the ZrSb_2_ structure. The general conclusion is that the presence of Ni atoms intensifies the interaction between Zr/Ni and Sb and, at the same time, reduces the bonding between Sb atoms. One may speculate that the composition of the Zr_3_NiSb_7_ compound may be the boundary limit of some solid solutions based on ZrSb_2_. However, the detailed study of the phase equilibria in the Zr–Ni–Sb system did not show a formation of any substitutional or interstitial solid solution. Moreover, the diffraction patterns of Zr_3_NiSb_7_ and ZrSb_2_ are rather different.

### Experimental

A sample with nominal composition Zr_30_Ni_10_Sb_60_ was prepared by arc-melting the constituent elements Zr (99.99 wt.%), Ni (99.99 wt.%), and Sb (99.99 wt.%) on a water-cooled copper hearth under a protective Ti-gettered argon atmosphere. 5 wt.% excess of Sb was required to compensate the evaporative loss during arc-melting. The ingot was annealed at 870 K for 720 h in an evacuated silica ampoule, and finally quenched in cold water. A crystal of the title compound suitable for single-crystal X-ray diffraction was extracted directly from the annealed sample. The chemical composition of the crystal was determined on the basis of an energy dispersive X-ray spectroscopical analysis using a Hitachi S-2700 scanning electron microscope. The result of the analysis is in good aggreement with the composition calculated from the structural refinement: Measured: 24.5 (8) %_at_ Zr, 11.3 (6) %_at_ Ni, 64.2 (16) %_at_ Sb; calculated Zr 27 %_at_, Ni 9%_at_, Sb 64 %_at_.

### Refinement

The highest remaining electron density peak and the deepest hole are located 0.80 Å from Sb1 and 1.78 Å from Ni1, respectively. The structure solution and refinement were also performed in the non-centrosymmetric space group *Pna*2_1_, but were less satisfactory and resulted in larger *R* indices and atomic displacement parameters.

### Crystal data

**Table:**

Zr_3_NiSb_7_	*F*_000_ = 2020
*M**_r_* = 1184.62	*D*_x_ = 7.946 Mg m^−^^3^
Orthorhombic, *P**n**m**a*	Mo *K*α radiation λ = 0.71073 Å
Hall symbol: -P 2ac 2n	Cell parameters from 4956 reflections
*a* = 17.5165 (19) Å	θ = 2.3–33.1º
*b* = 3.9266 (4) Å	µ = 23.56 mm^−^^1^
*c* = 14.3968 (15) Å	*T* = 295 (2) K
*V* = 990.22 (18) Å^3^	Needle, silver
*Z* = 4	0.37 × 0.06 × 0.04 mm

### Data collection

**Table:**

Bruker SMART 1000 diffractometer	1722 independent reflections
Radiation source: fine-focus sealed tube	1500 reflections with *I* > 2σ(*I*)
Monochromator: graphite	*R*_int_ = 0.044
*T* = 295(2) K	θ_max_ = 30.5º
φ and ω scans	θ_min_ = 2.3º
Absorption correction: numerical(SHELXTL; Sheldrick, 2008)	*h* = −25→25
*T*_min_ = 0.057, *T*_max_ = 0.426	*k* = −5→5
11118 measured reflections	*l* = −20→20

### Refinement

**Table:**

Refinement on *F*^2^	Secondary atom site location: difference Fourier map
Least-squares matrix: full	*w* = 1/[σ^2^(*F*_o_^2^) + (0.0223*P*)^2^ + 1.1518*P*] where *P* = (*F*_o_^2^ + 2*F*_c_^2^)/3
*R*[*F*^2^ > 2σ(*F*^2^)] = 0.024	(Δ/σ)_max_ = 0.001
*wR*(*F*^2^) = 0.054	Δρ_max_ = 2.12 e Å^−^^3^
*S* = 1.18	Δρ_min_ = −2.89 e Å^−^^3^
1722 reflections	Extinction correction: SHELXL97 (Sheldrick, 2008), Fc^*^=kFc[1+0.001xFc^2^λ^3^/sin(2θ)]^-1/4^
68 parameters	Extinction coefficient: 0.00069 (5)
Primary atom site location: structure-invariant direct methods	

### Special details

**Table:**

Geometry. All e.s.d.'s (except the e.s.d. in the dihedral angle between two l.s. planes) are estimated using the full covariance matrix. The cell e.s.d.'s are taken into account individually in the estimation of e.s.d.'s in distances, angles and torsion angles; correlations between e.s.d.'s in cell parameters are only used when they are defined by crystal symmetry. An approximate (isotropic) treatment of cell e.s.d.'s is used for estimating e.s.d.'s involving l.s. planes.
Refinement. Refinement of *F*^2^ against ALL reflections. The weighted *R*-factor *wR* and goodness of fit *S* are based on *F*^2^, conventional *R*-factors *R* are based on *F*, with *F* set to zero for negative *F*^2^. The threshold expression of *F*^2^ > σ(*F*^2^) is used only for calculating *R*-factors(gt) *etc*. and is not relevant to the choice of reflections for refinement. *R*-factors based on *F*^2^ are statistically about twice as large as those based on *F*, and *R*- factors based on ALL data will be even larger.

### Fractional atomic coordinates and isotropic or equivalent isotropic displacement parameters (Å^2^)

**Table:**

	*x*	*y*	*z*	*U*_iso_*/*U*_eq_	
Zr1	0.34664 (4)	0.2500	0.19076 (5)	0.00788 (14)	
Zr2	0.37045 (4)	0.2500	0.47072 (5)	0.00721 (13)	
Zr3	0.39237 (4)	0.2500	0.90990 (5)	0.00782 (14)	
Ni1	0.43680 (5)	0.2500	0.68908 (6)	0.00903 (18)	
Sb1	0.02147 (3)	0.2500	0.29766 (3)	0.00871 (11)	
Sb2	0.03748 (2)	0.2500	0.07626 (3)	0.00790 (10)	
Sb3	0.07123 (3)	0.2500	0.56057 (3)	0.00957 (11)	
Sb4	0.09131 (3)	0.2500	0.82504 (3)	0.00823 (10)	
Sb5	0.22833 (3)	0.2500	0.35390 (3)	0.00918 (11)	
Sb6	0.24792 (2)	0.2500	0.02153 (3)	0.00875 (11)	
Sb7	0.28995 (3)	0.2500	0.68532 (4)	0.01218 (11)	

### Atomic displacement parameters (Å^2^)

**Table:**

	*U*^11^	*U*^22^	*U*^33^	*U*^12^	*U*^13^	*U*^23^
Zr1	0.0068 (3)	0.0083 (3)	0.0085 (3)	0.000	−0.0007 (2)	0.000
Zr2	0.0054 (3)	0.0077 (3)	0.0085 (3)	0.000	0.0002 (2)	0.000
Zr3	0.0064 (3)	0.0078 (3)	0.0092 (3)	0.000	0.0004 (2)	0.000
Ni1	0.0085 (4)	0.0092 (5)	0.0094 (4)	0.000	0.0000 (3)	0.000
Sb1	0.0081 (2)	0.0089 (2)	0.0092 (2)	0.000	−0.00158 (15)	0.000
Sb2	0.0069 (2)	0.0084 (2)	0.0084 (2)	0.000	0.00046 (15)	0.000
Sb3	0.0104 (2)	0.0100 (2)	0.0083 (2)	0.000	0.00042 (16)	0.000
Sb4	0.0082 (2)	0.0086 (2)	0.0079 (2)	0.000	0.00022 (15)	0.000
Sb5	0.0069 (2)	0.0104 (2)	0.0102 (2)	0.000	0.00005 (15)	0.000
Sb6	0.0069 (2)	0.0100 (2)	0.0094 (2)	0.000	−0.00051 (15)	0.000
Sb7	0.0076 (2)	0.0100 (3)	0.0189 (3)	0.000	0.00142 (16)	0.000

### Geometric parameters (Å, °)

**Table:**

Zr1—Sb4^i^	2.9620 (6)	Sb2—Ni1^ii^	2.5876 (7)
Zr1—Sb4^ii^	2.9620 (6)	Sb2—Zr2^ii^	2.9604 (6)
Zr1—Sb6	2.9876 (8)	Sb2—Zr2^i^	2.9604 (6)
Zr1—Sb1^iii^	3.0669 (8)	Sb2—Zr2^viii^	3.0030 (8)
Zr1—Sb3^ii^	3.0720 (7)	Sb2—Sb2^xi^	3.2250 (7)
Zr1—Sb3^i^	3.0720 (7)	Sb2—Sb2^xii^	3.2250 (7)
Zr1—Sb7^ii^	3.0960 (6)	Sb2—Sb4^x^	3.3111 (6)
Zr1—Sb7^i^	3.0960 (6)	Sb2—Sb4^ix^	3.3111 (6)
Zr1—Sb5	3.1324 (8)	Sb3—Zr3^ii^	2.9943 (7)
Zr2—Sb6^iv^	2.9478 (6)	Sb3—Zr3^i^	2.9943 (7)
Zr2—Sb6^v^	2.9478 (6)	Sb3—Zr1^v^	3.0720 (7)
Zr2—Sb4^ii^	2.9499 (6)	Sb3—Zr1^iv^	3.0720 (7)
Zr2—Sb4^i^	2.9499 (6)	Sb3—Zr3^xiii^	3.1617 (9)
Zr2—Sb2^v^	2.9604 (6)	Sb3—Sb1^ix^	3.2645 (6)
Zr2—Sb2^iv^	2.9604 (6)	Sb3—Sb1^x^	3.2645 (6)
Zr2—Sb2^iii^	3.0029 (8)	Sb4—Ni1^xiii^	2.7141 (10)
Zr2—Sb5	3.0044 (8)	Sb4—Zr2^v^	2.9499 (6)
Zr2—Sb7	3.3962 (9)	Sb4—Zr2^iv^	2.9499 (6)
Zr3—Sb1^iv^	2.9569 (6)	Sb4—Zr1^iv^	2.9619 (6)
Zr3—Sb1^v^	2.9569 (6)	Sb4—Zr1^v^	2.9619 (6)
Zr3—Sb3^v^	2.9944 (7)	Sb4—Sb1^x^	3.2981 (6)
Zr3—Sb3^iv^	2.9944 (7)	Sb4—Sb1^ix^	3.2981 (6)
Zr3—Sb5^iv^	2.9958 (6)	Sb4—Sb2^x^	3.3111 (6)
Zr3—Sb5^v^	2.9958 (6)	Sb4—Sb2^ix^	3.3110 (6)
Zr3—Sb6^vi^	2.9975 (8)	Sb5—Zr3^ii^	2.9958 (6)
Zr3—Sb3^vii^	3.1616 (9)	Sb5—Zr3^i^	2.9958 (6)
Zr3—Ni1	3.2730 (12)	Sb5—Sb7^ii^	3.1380 (6)
Ni1—Sb7	2.5728 (10)	Sb5—Sb7^i^	3.1380 (6)
Ni1—Sb2^iv^	2.5875 (7)	Sb5—Sb6^v^	3.1387 (6)
Ni1—Sb2^v^	2.5875 (7)	Sb5—Sb6^iv^	3.1387 (6)
Ni1—Sb1^v^	2.6140 (7)	Sb6—Zr2^ii^	2.9478 (6)
Ni1—Sb1^iv^	2.6140 (7)	Sb6—Zr2^i^	2.9478 (6)
Ni1—Sb4^vii^	2.7141 (10)	Sb6—Zr3^xiv^	2.9974 (8)
Sb1—Ni1^ii^	2.6139 (7)	Sb6—Sb5^ii^	3.1388 (6)
Sb1—Ni1^i^	2.6139 (7)	Sb6—Sb5^i^	3.1388 (6)
Sb1—Zr3^i^	2.9570 (6)	Sb6—Sb7^i^	3.1393 (6)
Sb1—Zr3^ii^	2.9570 (6)	Sb6—Sb7^ii^	3.1393 (6)
Sb1—Zr1^viii^	3.0669 (8)	Sb7—Zr1^iv^	3.0960 (6)
Sb1—Sb2	3.1998 (8)	Sb7—Zr1^v^	3.0960 (6)
Sb1—Sb3^ix^	3.2645 (6)	Sb7—Sb5^v^	3.1380 (6)
Sb1—Sb3^x^	3.2645 (6)	Sb7—Sb5^iv^	3.1380 (6)
Sb1—Sb4^x^	3.2981 (6)	Sb7—Sb6^iv^	3.1393 (6)
Sb1—Sb4^ix^	3.2981 (6)	Sb7—Sb6^v^	3.1393 (6)
Sb2—Ni1^i^	2.5876 (7)		
			
Sb4^i^—Zr1—Sb4^ii^	83.03 (2)	Ni1^i^—Sb2—Zr2^viii^	108.12 (2)
Sb4^i^—Zr1—Sb6	138.128 (11)	Ni1^ii^—Sb2—Zr2^viii^	108.12 (2)
Sb4^ii^—Zr1—Sb6	138.128 (11)	Zr2^ii^—Sb2—Zr2^viii^	114.528 (17)
Sb4^i^—Zr1—Sb1^iii^	66.303 (16)	Zr2^i^—Sb2—Zr2^viii^	114.528 (17)
Sb4^ii^—Zr1—Sb1^iii^	66.303 (16)	Ni1^i^—Sb2—Sb1	52.409 (19)
Sb6—Zr1—Sb1^iii^	128.48 (3)	Ni1^ii^—Sb2—Sb1	52.409 (19)
Sb4^i^—Zr1—Sb3^ii^	130.54 (3)	Zr2^ii^—Sb2—Sb1	123.991 (17)
Sb4^ii^—Zr1—Sb3^ii^	78.623 (15)	Zr2^i^—Sb2—Sb1	123.991 (17)
Sb6—Zr1—Sb3^ii^	76.910 (19)	Zr2^viii^—Sb2—Sb1	97.986 (19)
Sb1^iii^—Zr1—Sb3^ii^	64.250 (16)	Ni1^i^—Sb2—Sb2^xi^	163.78 (3)
Sb4^i^—Zr1—Sb3^i^	78.623 (15)	Ni1^ii^—Sb2—Sb2^xi^	92.085 (17)
Sb4^ii^—Zr1—Sb3^i^	130.54 (3)	Zr2^ii^—Sb2—Sb2^xi^	57.900 (17)
Sb6—Zr1—Sb3^i^	76.911 (19)	Zr2^i^—Sb2—Sb2^xi^	106.03 (2)
Sb1^iii^—Zr1—Sb3^i^	64.250 (16)	Zr2^viii^—Sb2—Sb2^xi^	56.628 (16)
Sb3^ii^—Zr1—Sb3^i^	79.45 (2)	Sb1—Sb2—Sb2^xi^	129.982 (17)
Sb4^i^—Zr1—Sb7^ii^	136.09 (3)	Ni1^i^—Sb2—Sb2^xii^	92.085 (17)
Sb4^ii^—Zr1—Sb7^ii^	83.092 (15)	Ni1^ii^—Sb2—Sb2^xii^	163.78 (3)
Sb6—Zr1—Sb7^ii^	62.103 (16)	Zr2^ii^—Sb2—Sb2^xii^	106.03 (2)
Sb1^iii^—Zr1—Sb7^ii^	140.627 (10)	Zr2^i^—Sb2—Sb2^xii^	57.900 (16)
Sb3^ii^—Zr1—Sb7^ii^	86.630 (15)	Zr2^viii^—Sb2—Sb2^xii^	56.628 (16)
Sb3^i^—Zr1—Sb7^ii^	138.75 (3)	Sb1—Sb2—Sb2^xii^	129.982 (17)
Sb4^i^—Zr1—Sb7^i^	83.092 (15)	Sb2^xi^—Sb2—Sb2^xii^	75.00 (2)
Sb4^ii^—Zr1—Sb7^i^	136.09 (3)	Ni1^i^—Sb2—Sb4^x^	107.40 (3)
Sb6—Zr1—Sb7^i^	62.103 (16)	Ni1^ii^—Sb2—Sb4^x^	53.08 (2)
Sb1^iii^—Zr1—Sb7^i^	140.627 (10)	Zr2^ii^—Sb2—Sb4^x^	101.431 (12)
Sb3^ii^—Zr1—Sb7^i^	138.75 (3)	Zr2^i^—Sb2—Sb4^x^	169.95 (2)
Sb3^i^—Zr1—Sb7^i^	86.630 (15)	Zr2^viii^—Sb2—Sb4^x^	55.446 (14)
Sb7^ii^—Zr1—Sb7^i^	78.71 (2)	Sb1—Sb2—Sb4^x^	60.840 (12)
Sb4^i^—Zr1—Sb5	75.722 (19)	Sb2^xi^—Sb2—Sb4^x^	69.745 (15)
Sb4^ii^—Zr1—Sb5	75.722 (19)	Sb2^xii^—Sb2—Sb4^x^	112.07 (2)
Sb6—Zr1—Sb5	103.21 (2)	Ni1^i^—Sb2—Sb4^ix^	53.08 (2)
Sb1^iii^—Zr1—Sb5	128.31 (3)	Ni1^ii^—Sb2—Sb4^ix^	107.40 (3)
Sb3^ii^—Zr1—Sb5	140.115 (11)	Zr2^ii^—Sb2—Sb4^ix^	169.95 (2)
Sb3^i^—Zr1—Sb5	140.115 (11)	Zr2^i^—Sb2—Sb4^ix^	101.431 (12)
Sb7^ii^—Zr1—Sb5	60.504 (16)	Zr2^viii^—Sb2—Sb4^ix^	55.446 (14)
Sb7^i^—Zr1—Sb5	60.504 (16)	Sb1—Sb2—Sb4^ix^	60.840 (12)
Sb6^iv^—Zr2—Sb6^v^	83.52 (2)	Sb2^xi^—Sb2—Sb4^ix^	112.07 (2)
Sb6^iv^—Zr2—Sb4^ii^	83.851 (14)	Sb2^xii^—Sb2—Sb4^ix^	69.745 (15)
Sb6^v^—Zr2—Sb4^ii^	141.21 (3)	Sb4^x^—Sb2—Sb4^ix^	72.732 (15)
Sb6^iv^—Zr2—Sb4^i^	141.21 (3)	Zr3^ii^—Sb3—Zr3^i^	81.94 (2)
Sb6^v^—Zr2—Sb4^i^	83.851 (14)	Zr3^ii^—Sb3—Zr1^v^	139.58 (2)
Sb4^ii^—Zr2—Sb4^i^	83.45 (2)	Zr3^i^—Sb3—Zr1^v^	85.597 (17)
Sb6^iv^—Zr2—Sb2^v^	134.23 (3)	Zr3^ii^—Sb3—Zr1^iv^	85.597 (16)
Sb6^v^—Zr2—Sb2^v^	79.287 (15)	Zr3^i^—Sb3—Zr1^iv^	139.58 (2)
Sb4^ii^—Zr2—Sb2^v^	133.05 (3)	Zr1^v^—Sb3—Zr1^iv^	79.45 (2)
Sb4^i^—Zr2—Sb2^v^	78.456 (15)	Zr3^ii^—Sb3—Zr3^xiii^	107.962 (18)
Sb6^iv^—Zr2—Sb2^iv^	79.287 (15)	Zr3^i^—Sb3—Zr3^xiii^	107.962 (18)
Sb6^v^—Zr2—Sb2^iv^	134.23 (3)	Zr1^v^—Sb3—Zr3^xiii^	112.458 (19)
Sb4^ii^—Zr2—Sb2^iv^	78.456 (15)	Zr1^iv^—Sb3—Zr3^xiii^	112.458 (19)
Sb4^i^—Zr2—Sb2^iv^	133.05 (3)	Zr3^ii^—Sb3—Sb1^ix^	162.39 (2)
Sb2^v^—Zr2—Sb2^iv^	83.09 (2)	Zr3^i^—Sb3—Sb1^ix^	99.468 (13)
Sb6^iv^—Zr2—Sb2^iii^	137.839 (11)	Zr1^v^—Sb3—Sb1^ix^	57.799 (16)
Sb6^v^—Zr2—Sb2^iii^	137.839 (11)	Zr1^iv^—Sb3—Sb1^ix^	103.64 (2)
Sb4^ii^—Zr2—Sb2^iii^	67.583 (16)	Zr3^xiii^—Sb3—Sb1^ix^	54.764 (13)
Sb4^i^—Zr2—Sb2^iii^	67.583 (16)	Zr3^ii^—Sb3—Sb1^x^	99.468 (13)
Sb2^v^—Zr2—Sb2^iii^	65.474 (17)	Zr3^i^—Sb3—Sb1^x^	162.39 (2)
Sb2^iv^—Zr2—Sb2^iii^	65.474 (17)	Zr1^v^—Sb3—Sb1^x^	103.64 (2)
Sb6^iv^—Zr2—Sb5	63.641 (17)	Zr1^iv^—Sb3—Sb1^x^	57.799 (15)
Sb6^v^—Zr2—Sb5	63.641 (17)	Zr3^xiii^—Sb3—Sb1^x^	54.764 (13)
Sb4^ii^—Zr2—Sb5	77.886 (19)	Sb1^ix^—Sb3—Sb1^x^	73.942 (15)
Sb4^i^—Zr2—Sb5	77.886 (19)	Ni1^xiii^—Sb4—Zr2^v^	106.24 (2)
Sb2^v^—Zr2—Sb5	137.621 (11)	Ni1^xiii^—Sb4—Zr2^iv^	106.24 (2)
Sb2^iv^—Zr2—Sb5	137.621 (12)	Zr2^v^—Sb4—Zr2^iv^	83.45 (2)
Sb2^iii^—Zr2—Sb5	132.94 (3)	Ni1^xiii^—Sb4—Zr1^iv^	108.49 (2)
Sb6^iv^—Zr2—Sb7	58.813 (15)	Zr2^v^—Sb4—Zr1^iv^	145.27 (2)
Sb6^v^—Zr2—Sb7	58.813 (15)	Zr2^iv^—Sb4—Zr1^iv^	86.535 (17)
Sb4^ii^—Zr2—Sb7	137.823 (12)	Ni1^xiii^—Sb4—Zr1^v^	108.49 (2)
Sb4^i^—Zr2—Sb7	137.823 (12)	Zr2^v^—Sb4—Zr1^v^	86.535 (17)
Sb2^v^—Zr2—Sb7	76.068 (18)	Zr2^iv^—Sb4—Zr1^v^	145.27 (2)
Sb2^iv^—Zr2—Sb7	76.068 (18)	Zr1^iv^—Sb4—Zr1^v^	83.03 (2)
Sb2^iii^—Zr2—Sb7	127.55 (2)	Ni1^xiii^—Sb4—Sb1^x^	50.403 (16)
Sb5—Zr2—Sb7	99.51 (2)	Zr2^v^—Sb4—Sb1^x^	155.92 (2)
Sb1^iv^—Zr3—Sb1^v^	83.21 (2)	Zr2^iv^—Sb4—Sb1^x^	96.928 (13)
Sb1^iv^—Zr3—Sb3^v^	136.27 (3)	Zr1^iv^—Sb4—Sb1^x^	58.375 (16)
Sb1^v^—Zr3—Sb3^v^	81.481 (15)	Zr1^v^—Sb4—Sb1^x^	105.36 (2)
Sb1^iv^—Zr3—Sb3^iv^	81.481 (16)	Ni1^xiii^—Sb4—Sb1^ix^	50.403 (16)
Sb1^v^—Zr3—Sb3^iv^	136.27 (3)	Zr2^v^—Sb4—Sb1^ix^	96.928 (13)
Sb3^v^—Zr3—Sb3^iv^	81.94 (2)	Zr2^iv^—Sb4—Sb1^ix^	155.92 (2)
Sb1^iv^—Zr3—Sb5^iv^	77.172 (16)	Zr1^iv^—Sb4—Sb1^ix^	105.36 (2)
Sb1^v^—Zr3—Sb5^iv^	130.41 (3)	Zr1^v^—Sb4—Sb1^ix^	58.375 (16)
Sb3^v^—Zr3—Sb5^iv^	140.80 (3)	Sb1^x^—Sb4—Sb1^ix^	73.066 (15)
Sb3^iv^—Zr3—Sb5^iv^	85.155 (14)	Ni1^xiii^—Sb4—Sb2^x^	49.662 (16)
Sb1^iv^—Zr3—Sb5^v^	130.41 (3)	Zr2^v^—Sb4—Sb2^x^	104.14 (2)
Sb1^v^—Zr3—Sb5^v^	77.172 (15)	Zr2^iv^—Sb4—Sb2^x^	56.972 (16)
Sb3^v^—Zr3—Sb5^v^	85.155 (14)	Zr1^iv^—Sb4—Sb2^x^	97.880 (13)
Sb3^iv^—Zr3—Sb5^v^	140.80 (3)	Zr1^v^—Sb4—Sb2^x^	157.39 (2)
Sb5^iv^—Zr3—Sb5^v^	81.89 (2)	Sb1^x^—Sb4—Sb2^x^	57.912 (14)
Sb1^iv^—Zr3—Sb6^vi^	136.371 (13)	Sb1^ix^—Sb4—Sb2^x^	100.063 (17)
Sb1^v^—Zr3—Sb6^vi^	136.371 (13)	Ni1^xiii^—Sb4—Sb2^ix^	49.662 (16)
Sb3^v^—Zr3—Sb6^vi^	77.956 (19)	Zr2^v^—Sb4—Sb2^ix^	56.972 (16)
Sb3^iv^—Zr3—Sb6^vi^	77.956 (19)	Zr2^iv^—Sb4—Sb2^ix^	104.14 (2)
Sb5^iv^—Zr3—Sb6^vi^	63.164 (17)	Zr1^iv^—Sb4—Sb2^ix^	157.39 (2)
Sb5^v^—Zr3—Sb6^vi^	63.164 (17)	Zr1^v^—Sb4—Sb2^ix^	97.880 (13)
Sb1^iv^—Zr3—Sb3^vii^	64.388 (16)	Sb1^x^—Sb4—Sb2^ix^	100.063 (17)
Sb1^v^—Zr3—Sb3^vii^	64.388 (16)	Sb1^ix^—Sb4—Sb2^ix^	57.912 (14)
Sb3^v^—Zr3—Sb3^vii^	72.038 (18)	Sb2^x^—Sb4—Sb2^ix^	72.734 (15)
Sb3^iv^—Zr3—Sb3^vii^	72.038 (18)	Zr3^ii^—Sb5—Zr3^i^	81.89 (2)
Sb5^iv^—Zr3—Sb3^vii^	137.351 (12)	Zr3^ii^—Sb5—Zr2	115.73 (2)
Sb5^v^—Zr3—Sb3^vii^	137.351 (12)	Zr3^i^—Sb5—Zr2	115.73 (2)
Sb6^vi^—Zr3—Sb3^vii^	139.85 (3)	Zr3^ii^—Sb5—Zr1	131.970 (16)
Sb1^iv^—Zr3—Ni1	49.295 (15)	Zr3^i^—Sb5—Zr1	131.970 (16)
Sb1^v^—Zr3—Ni1	49.295 (15)	Zr2—Sb5—Zr1	82.62 (2)
Sb3^v^—Zr3—Ni1	130.769 (18)	Zr3^ii^—Sb5—Sb7^ii^	74.105 (16)
Sb3^iv^—Zr3—Ni1	130.769 (18)	Zr3^i^—Sb5—Sb7^ii^	123.11 (2)
Sb5^iv^—Zr3—Ni1	84.63 (2)	Zr2—Sb5—Sb7^ii^	121.163 (17)
Sb5^v^—Zr3—Ni1	84.63 (2)	Zr1—Sb5—Sb7^ii^	59.174 (15)
Sb6^vi^—Zr3—Ni1	136.18 (3)	Zr3^ii^—Sb5—Sb7^i^	123.11 (2)
Sb3^vii^—Zr3—Ni1	83.97 (2)	Zr3^i^—Sb5—Sb7^i^	74.105 (16)
Sb7—Ni1—Sb2^iv^	99.26 (3)	Zr2—Sb5—Sb7^i^	121.163 (17)
Sb7—Ni1—Sb2^v^	99.26 (3)	Zr1—Sb5—Sb7^i^	59.174 (15)
Sb2^iv^—Ni1—Sb2^v^	98.71 (3)	Sb7^ii^—Sb5—Sb7^i^	77.460 (18)
Sb7—Ni1—Sb1^v^	106.99 (3)	Zr3^ii^—Sb5—Sb6^v^	107.25 (2)
Sb2^iv^—Ni1—Sb1^v^	153.71 (4)	Zr3^i^—Sb5—Sb6^v^	58.444 (16)
Sb2^v^—Ni1—Sb1^v^	75.925 (16)	Zr2—Sb5—Sb6^v^	57.301 (14)
Sb7—Ni1—Sb1^iv^	106.99 (3)	Zr1—Sb5—Sb6^v^	119.266 (16)
Sb2^iv^—Ni1—Sb1^iv^	75.925 (16)	Sb7^ii^—Sb5—Sb6^v^	178.23 (2)
Sb2^v^—Ni1—Sb1^iv^	153.71 (4)	Sb7^i^—Sb5—Sb6^v^	102.523 (11)
Sb1^v^—Ni1—Sb1^iv^	97.37 (3)	Zr3^ii^—Sb5—Sb6^iv^	58.444 (16)
Sb7—Ni1—Sb4^vii^	174.50 (4)	Zr3^i^—Sb5—Sb6^iv^	107.25 (2)
Sb2^iv^—Ni1—Sb4^vii^	77.26 (2)	Zr2—Sb5—Sb6^iv^	57.302 (14)
Sb2^v^—Ni1—Sb4^vii^	77.26 (2)	Zr1—Sb5—Sb6^iv^	119.266 (16)
Sb1^v^—Ni1—Sb4^vii^	76.46 (2)	Sb7^ii^—Sb5—Sb6^iv^	102.523 (11)
Sb1^iv^—Ni1—Sb4^vii^	76.46 (2)	Sb7^i^—Sb5—Sb6^iv^	178.23 (2)
Sb7—Ni1—Zr3	77.45 (3)	Sb6^v^—Sb5—Sb6^iv^	77.438 (18)
Sb2^iv^—Ni1—Zr3	130.623 (17)	Zr2^ii^—Sb6—Zr2^i^	83.52 (2)
Sb2^v^—Ni1—Zr3	130.623 (17)	Zr2^ii^—Sb6—Zr1	127.554 (17)
Sb1^v^—Ni1—Zr3	59.04 (2)	Zr2^i^—Sb6—Zr1	127.554 (17)
Sb1^iv^—Ni1—Zr3	59.04 (2)	Zr2^ii^—Sb6—Zr3^xiv^	117.435 (19)
Sb4^vii^—Ni1—Zr3	108.05 (3)	Zr2^i^—Sb6—Zr3^xiv^	117.435 (19)
Ni1^ii^—Sb1—Ni1^i^	97.37 (3)	Zr1—Sb6—Zr3^xiv^	87.06 (2)
Ni1^ii^—Sb1—Zr3^i^	133.06 (3)	Zr2^ii^—Sb6—Sb5^ii^	59.058 (16)
Ni1^i^—Sb1—Zr3^i^	71.66 (2)	Zr2^i^—Sb6—Sb5^ii^	108.60 (2)
Ni1^ii^—Sb1—Zr3^ii^	71.66 (2)	Zr1—Sb6—Sb5^ii^	123.387 (17)
Ni1^i^—Sb1—Zr3^ii^	133.06 (3)	Zr3^xiv^—Sb6—Sb5^ii^	58.391 (14)
Zr3^i^—Sb1—Zr3^ii^	83.21 (2)	Zr2^ii^—Sb6—Sb5^i^	108.60 (2)
Ni1^ii^—Sb1—Zr1^viii^	108.17 (2)	Zr2^i^—Sb6—Sb5^i^	59.058 (16)
Ni1^i^—Sb1—Zr1^viii^	108.17 (2)	Zr1—Sb6—Sb5^i^	123.387 (17)
Zr3^i^—Sb1—Zr1^viii^	118.681 (19)	Zr3^xiv^—Sb6—Sb5^i^	58.391 (14)
Zr3^ii^—Sb1—Zr1^viii^	118.681 (19)	Sb5^ii^—Sb6—Sb5^i^	77.437 (18)
Ni1^ii^—Sb1—Sb2	51.665 (19)	Zr2^ii^—Sb6—Sb7^i^	117.02 (2)
Ni1^i^—Sb1—Sb2	51.665 (19)	Zr2^i^—Sb6—Sb7^i^	67.743 (16)
Zr3^i^—Sb1—Sb2	119.976 (18)	Zr1—Sb6—Sb7^i^	60.643 (16)
Zr3^ii^—Sb1—Sb2	119.976 (18)	Zr3^xiv^—Sb6—Sb7^i^	125.528 (15)
Zr1^viii^—Sb1—Sb2	98.147 (19)	Sb5^ii^—Sb6—Sb7^i^	175.37 (2)
Ni1^ii^—Sb1—Sb3^ix^	164.74 (2)	Sb5^i^—Sb6—Sb7^i^	102.379 (11)
Ni1^i^—Sb1—Sb3^ix^	93.514 (18)	Zr2^ii^—Sb6—Sb7^ii^	67.743 (16)
Zr3^i^—Sb1—Sb3^ix^	60.848 (17)	Zr2^i^—Sb6—Sb7^ii^	117.02 (2)
Zr3^ii^—Sb1—Sb3^ix^	108.15 (2)	Zr1—Sb6—Sb7^ii^	60.643 (16)
Zr1^viii^—Sb1—Sb3^ix^	57.951 (14)	Zr3^xiv^—Sb6—Sb7^ii^	125.528 (15)
Sb2—Sb1—Sb3^ix^	131.792 (14)	Sb5^ii^—Sb6—Sb7^ii^	102.379 (11)
Ni1^ii^—Sb1—Sb3^x^	93.514 (18)	Sb5^i^—Sb6—Sb7^ii^	175.37 (2)
Ni1^i^—Sb1—Sb3^x^	164.74 (2)	Sb7^i^—Sb6—Sb7^ii^	77.422 (18)
Zr3^i^—Sb1—Sb3^x^	108.15 (2)	Ni1—Sb7—Zr1^iv^	140.543 (10)
Zr3^ii^—Sb1—Sb3^x^	60.848 (17)	Ni1—Sb7—Zr1^v^	140.543 (10)
Zr1^viii^—Sb1—Sb3^x^	57.951 (14)	Zr1^iv^—Sb7—Zr1^v^	78.71 (2)
Sb2—Sb1—Sb3^x^	131.792 (13)	Ni1—Sb7—Sb5^v^	94.92 (2)
Sb3^ix^—Sb1—Sb3^x^	73.942 (16)	Zr1^iv^—Sb7—Sb5^v^	107.36 (2)
Ni1^ii^—Sb1—Sb4^x^	53.14 (2)	Zr1^v^—Sb7—Sb5^v^	60.321 (16)
Ni1^i^—Sb1—Sb4^x^	107.12 (3)	Ni1—Sb7—Sb5^iv^	94.92 (2)
Zr3^i^—Sb1—Sb4^x^	173.544 (17)	Zr1^iv^—Sb7—Sb5^iv^	60.321 (16)
Zr3^ii^—Sb1—Sb4^x^	101.722 (12)	Zr1^v^—Sb7—Sb5^iv^	107.36 (2)
Zr1^viii^—Sb1—Sb4^x^	55.321 (14)	Sb5^v^—Sb7—Sb5^iv^	77.460 (18)
Sb2—Sb1—Sb4^x^	61.249 (13)	Ni1—Sb7—Sb6^iv^	103.12 (2)
Sb3^ix^—Sb1—Sb4^x^	113.263 (18)	Zr1^iv^—Sb7—Sb6^iv^	57.253 (16)
Sb3^x^—Sb1—Sb4^x^	71.272 (14)	Zr1^v^—Sb7—Sb6^iv^	104.61 (2)
Ni1^ii^—Sb1—Sb4^ix^	107.12 (3)	Sb5^v^—Sb7—Sb6^iv^	161.93 (2)
Ni1^i^—Sb1—Sb4^ix^	53.14 (2)	Sb5^iv^—Sb7—Sb6^iv^	99.677 (12)
Zr3^i^—Sb1—Sb4^ix^	101.722 (12)	Ni1—Sb7—Sb6^v^	103.12 (2)
Zr3^ii^—Sb1—Sb4^ix^	173.544 (17)	Zr1^iv^—Sb7—Sb6^v^	104.61 (2)
Zr1^viii^—Sb1—Sb4^ix^	55.321 (14)	Zr1^v^—Sb7—Sb6^v^	57.253 (16)
Sb2—Sb1—Sb4^ix^	61.249 (13)	Sb5^v^—Sb7—Sb6^v^	99.677 (12)
Sb3^ix^—Sb1—Sb4^ix^	71.272 (14)	Sb5^iv^—Sb7—Sb6^v^	161.93 (2)
Sb3^x^—Sb1—Sb4^ix^	113.263 (18)	Sb6^iv^—Sb7—Sb6^v^	77.422 (18)
Sb4^x^—Sb1—Sb4^ix^	73.066 (15)	Ni1—Sb7—Zr2	66.67 (2)
Ni1^i^—Sb2—Ni1^ii^	98.71 (3)	Zr1^iv^—Sb7—Zr2	110.11 (2)
Ni1^i^—Sb2—Zr2^ii^	136.93 (3)	Zr1^v^—Sb7—Zr2	110.11 (2)
Ni1^ii^—Sb2—Zr2^ii^	73.99 (2)	Sb5^v^—Sb7—Zr2	138.240 (11)
Ni1^i^—Sb2—Zr2^i^	73.99 (2)	Sb5^iv^—Sb7—Zr2	138.240 (11)
Ni1^ii^—Sb2—Zr2^i^	136.93 (3)	Sb6^iv^—Sb7—Zr2	53.446 (14)
Zr2^ii^—Sb2—Zr2^i^	83.09 (2)	Sb6^v^—Sb7—Zr2	53.446 (14)

Symmetry codes: (i) −*x*+1/2, −*y*+1, *z*−1/2; (ii) −*x*+1/2, −*y*, *z*−1/2; (iii) *x*+1/2, *y*, −*z*+1/2; (iv) −*x*+1/2, −*y*, *z*+1/2; (v) −*x*+1/2, −*y*+1, *z*+1/2; (vi) *x*, *y*, *z*+1; (vii) *x*+1/2, *y*, −*z*+3/2; (viii) *x*−1/2, *y*, −*z*+1/2; (ix) −*x*, −*y*+1, −*z*+1; (x) −*x*, −*y*, −*z*+1; (xi) −*x*, −*y*, −*z*; (xii) −*x*, −*y*+1, −*z*; (xiii) *x*−1/2, *y*, −*z*+3/2; (xiv) *x*, *y*, *z*−1.
